# 'A limpet on a ship': Spatio‐temporal dynamics of patient and public involvement in research

**DOI:** 10.1111/hex.13215

**Published:** 2021-03-21

**Authors:** Stan (Constantina) Papoulias, Felicity Callard

**Affiliations:** ^1^ King’s College London London UK; ^2^ University of Glasgow Glasgow Scotland

**Keywords:** anthropology of meetings, applied health research, ethnography, mental health, patient and public involvement (PPI)

## Abstract

**Objective:**

To understand how current funding expectations that applied health research is undertaken in partnership with research institutions, health service providers and other stakeholders may impact on patient and public involvement (PPI).

**Background:**

While there is considerable research on the potential impact of PPI in health research, the processes of embedding PPI in research teams remain understudied. We draw on anthropological research on meetings as sites of production and reproduction of institutional cultures and external contexts to investigate how these functions of meetings may affect the potential contributions of patients, carers and the public in research.

**Methods:**

We present an ethnography of meetings that draws from a larger set of case studies of PPI in applied health research settings. The study draws on ethnographic observations, interviews with team members, analysis of documents and a presentation of preliminary findings through which feedback from informants was gathered.

**Results:**

We identified four means by which the oversight meetings regulated research and constrained the possibilities for PPI: a logic of ‘deliverables’ and imagined interlocutors, the performance of inclusion, positioning PPI in an ‘elsewhere’ of research, and the use of meetings to embed apprenticeship for junior researchers.

**Conclusions:**

PPI is essentially out of sync from the institutional logic of ‘deliverables’ constituting research partnerships. Embedding PPI in research requires challenging this logic.

## 1 INTRODUCTION: THE MISSING JIGSAW PIECE

*‘*You’ve got lots of different people around with [...] different areas that they’re … fabulously good at and have lots of different kinds of expertise. You’ve got your statistician, you’ve got your person with lived experience, you’ve got [someone who] knows the nuances of clinical trial bureaucracy and … somebody who’s good at … thinking out research questions, and somebody who’s good at solving practical problems …. It’s another piece of that *teamwork jigsaw* that’s important.’

B – Clinical academic, Health in Mind project, italics added

‘[quality improvement without patient involvement] it’s like a jigsaw and you’ve got a missing piece, you haven’t got the whole picture …’ (Quotation 1, Interviewee E, Quality improvement manager, italics added)^1^

Patient and public involvement (PPI) in health research positions patients and members of the public as actors undertaking or contributing to research rather than simply as its recipients or beneficiaries.^2^ Yet, while there is considerable literature on PPI in research and quality improvement,^3^, ^4^, ^5^, ^6^ as well as on how different modalities of collaboration organize knowledge production,^7^, ^8^, ^9^ little explicit attention has been paid to how the choreography and performance of collaborative research affects how PPI in particular is imagined and practised.^10^, ^11^ Thompson, and subsequently Vermeulen, have deployed the term ‘choreography’ to demonstrate and understand how aspects of the world commonly thought to belong to ‘different ontological orders’ come together.^12^, ^13^ We use choreography to think through how collaborative projects involving PPI attempt to hold together different ways of doing research and envisaging expertise. They bring together heterogeneous people, infrastructures and technologies (such as meetings and minutes); the manner in which they do so – the kinds of sequences and spatial dynamics that unfold – end up centring certain people and priorities at the same time as pushing others to the margins. Understanding such temporal and spatial processes is particularly important at a moment in which PPI is being consolidated within highly regulated and governed collaborative settings, such as funded health research, beholden to numerous stakeholders and conditioned by tight timelines.^14^

How PPI is practised – how choreographies unfold – may differ starkly from how PPI is commonly imagined by various stakeholders. For example, we have been struck by how frequently the figure of the jigsaw puzzle appears in descriptions of research involving PPI. Here, people with lived experience comprise the ‘missing piece’, which promises access to an epistemological and ontological ‘whole picture’. In the first example, above, drawn from an interview with a clinical academic from the study discussed in this paper, health research is presented as a collaborative practice in which all kinds of expertise smoothly join together: knowledge from ‘lived experience’ sits snugly alongside statistical reasoning and clinical trial bureaucracy. Such an imaginary conjures a harmonious choreography – one where all actors have a part and move forward together. But this is not the imaginary that materialized through the ethnography we present here. In this paper, we depart from the figure of the jigsaw puzzle to arrive at the alternative figure of the ‘limpet on the ship’. In so doing, we address the disjuncture between common spatial and temporal imaginaries of PPI within research and practices of collaborative research which position PPI spatially and temporally out of synch with other parts of the research endeavour.^15^ In our ethnographic analysis of meetings overseeing interlinked applied health research projects, we are concerned both with how those participating in PPI conceive of PPI and with how the logics that govern collaborative health research – logics that unfold through establishing an order that governs what needs to happen, when and in relation to whom – end up shaping practices of PPI. We explore how research involving PPI ends up framing where PPI belongs and how this has a significant effect on the epistemic contributions that PPI representatives (the term we use throughout) are able to make.

Data are drawn from a larger, comparative ethnographic study which investigated PPI in different applied health research projects, funded through a regionally awarded infrastructure grant from the UK’s National Institute for Health Research (NIHR). Infrastructure grants provide a five‐year funding cycle for research where the emphasis is on the development of partnerships between regional and national stakeholders – including NHS Trusts, local government, commissioners, industry, charities and service users and the public. While PPI in research is now mandatory for NIHR and other major UK funders, its role is particularly important in the case of infrastructure grants, whose very objective is the development of sustainable collaborations. While all research environments are collaborative, in infrastructure grants, partnership is not a means to an end but rather it itself *is the end, or outcome*. Therefore, our examination of how PPI is enacted in these grants also opens up broader questions concerning the staging and performance of collaboration within applied health research, especially in an environment increasingly driven by consumerist, managerial and performance‐focused logics, accountability requirements and practices.^16^, ^17^

## 2 MEETINGS AS A SOCIAL FORM

Team meetings are indispensable elements of collaborative scientific and health research, and, in addition, the steering committee or advisory group remains arguably the most common site for PPI.^18^ However, the dynamics of meetings and their role in embedding PPI remain understudied. While qualitative studies of PPI routinely include observations of meetings,^19^ it is often interviews, and their retrospective discussion of meetings, which take centre stage. When studies do focus on meetings themselves, they often consider these as sites for decision making and borrow approaches from management studies and organizational psychology, which seek to optimize meeting effectiveness, transparency and accountability and explore barriers to equitable participation.^20^ O'Shea and colleagues’ ethnography takes a more sociological approach, considering how lay input to an NHS Clinical Commissioning Group was constrained by social and professional stratifications which defined the parameters of control over decision making.^21^ Komporozos‐Athanasiou and colleagues’ ethnographic research uncovers the ‘powerful ritual structures’ of meetings that ‘serve to legitimate policy‐endorsed PPI aims and neutralize divergence from those aims’.^11^ Martin and Finn, indebted to the sociology of team work, consider meetings as sites of ‘habitual immersion’ through which ‘team members’ become acculturated to institutional culture which works to reproduce dominant power relations.^10^ Renedo and Marston's extensive ethnography of participatory quality improvement work in an NIHR infrastructure grant setting^22^ focuses on the objects and formal conventions that enable such ‘habitual immersion’ – showing how these facilitate PPI representatives’ alignment with what professionals ‘want’.^1^ They also demonstrate how the social, material and temporal dimensions of participatory spaces both shape how PPI takes place and are shaped by PPI representatives’ making and remaking of relations and interconnections.^22^

The approach to meetings we take here, while drawing on these literatures, is particularly indebted to Helen B. Schwartzman, whose foundational anthropological work has been extended by other anthropologists of meetings.^23^, ^24^, ^25^, ^26^ Schwartzman was central in the turn away from decision‐centric approaches – instead defining the meeting as a ‘pervasive social form’ which ‘produces and reproduces power relations and systems of control’. Here, meetings enact the norms of an organization/group: through the repetition of particular, conventionalized acts, the organization/group, and the participants’ membership in it, are both brought into being.^27^ Meetings are time‐bound interactions whose tacit rules constrain what are (in)appropriate uses of such time (what is on/off topic, who speaks and how, what actions should result from the meeting). Importantly, meetings are not self‐contained events, but refer to objects *beyond* themselves (eg the organization, other stakeholders). The anthropology of meetings invites us to consider how, by redirecting attention towards their anticipated effect on distant sites and a ‘future organizational imaginary’, meetings make their mundane rituals invisible.^25^ An anthropology of meetings removes this invisibility and considers instead the opaqueness of the rituals which constitute them and of the objects which mediate their interactions.^24^

## 3 SETTING AND METHODS

This paper discusses one aspect of a broader ethnography of PPI on a project we call Health in Mind (HiM) (a feasibility randomized controlled trial of a psychosocial intervention designed to encourage people with a psychiatric diagnosis to improve their physical health). The case study involved the first author, SP, undertaking extensive fieldwork from November 2015 to September 2017, which included 53 hours of observations of both regular and occasional meetings and of aspects of the delivery of the interventions as well as several informal exchanges with team members before or during observations. SP also conducted 17 semi‐structured interviews (lasting 40–70 minutes) with most team members (senior clinicians sharing oversight of HiM and interlinked projects, researchers responsible for running the project, health‐care workers running interventions, a lay volunteer working in parts of the intervention and two PPI representatives). The questions for many of the interviews were informed by observations of the meetings, as well as by literature on PPI. The interviews were digitally recorded and transcribed verbatim while SP took extensive fieldnotes both during and in the immediate aftermath of observations. All study participants provided written informed consent and were given the opportunity to withdraw at any time. In the case of the meetings and informal exchanges specifically, fieldnotes were taken only if every participant had given consent. Additionally, SP analysed 67 documents – meeting minutes and agendas, progress summaries, PowerPoint presentations, emails and study protocols. After the completion of observations, SP organized a 90‐minute workshop in which participants were invited to hear preliminary findings and to reflect on PPI in the study. The workshop was digitally recorded and transcribed verbatim.

SP analysed the material by drawing the fieldnotes, interviews, documents and workshop transcript into one dataset, reading all materials multiple times and developing codes thematically that could move across data gathered from different phases.^28^ Analysis and interpretation proceeded both inductively (by finding meaning within the data) and through reference to problematics identified in the literatures on PPI and the anthropology of meetings. The second author (FC) acted as interlocutor – assisting in refining codes and developing the themes presented in the findings. We have maintained anonymity by changing/omitting certain participant details and using initials of pseudonyms approved by participants. The study secured a favourable opinion from the East of Scotland Research Ethics Service15/ES/0162. Here, we focus on the staging of PPI through the meetings of a steering group that oversaw both HiM and interlinked projects.

## 4 FINDINGS

We first summarize the general character of the meetings so that readers can orient themselves in relation to four overarching themes that comprise our findings: (i) the spatio‐temporal logic of deliverables; (ii) maintaining the appearance of inclusion; (iii) installing PPI as a constitutive ‘elsewhere’; and (iv) acculturating junior researchers: meetings as sites of apprenticeship.

The observed meetings were monthly, lasting 60–90 minutes. Their stated purpose was to provide oversight on a workstream of inter‐related, small‐scale projects. These were of varying design and sought to align university‐led research to local NHS Trust priorities and directives: integrating physical and mental health care and supporting mental health service users in managing physical health. The oversight consisted in monitoring progress and advising on potential challenges to the running of studies. Initially, meetings were small, consisting in two senior clinical academics with roles in the partnered NHS Trust, who were lead investigators, and two early/mid‐career researchers on fixed‐term contracts. After six months, meetings broadened to include the leads and research workers of five additional projects incorporated in the workstream, as well as an administrator. Yet, they remained small in‐house affairs, typically involving approximately 8 people working in adjacent university buildings, although trust managers and clinicians were occasionally invited. Two PPI representatives, a service user and a carer, were invited to join the meetings at an early stage and on a quarterly basis (after mutual agreement concerning their availability). The two had an on‐going relationship with the senior staff, familiarity with research processes, a declared interest in physical health management and had been specifically invited to provide advice and oversight on HiM. However, since in effect these meetings provided joint oversight, the representatives engaged with all eight projects.

### 4.1 The spatio‐temporal logic of deliverables

#### 4.1.1 Conjuring absent interlocutors

Since the meetings’ purpose was to ensure projects ran successfully to completion, meetings were constituted as a site for rehearsing responses to the demands of conjured interlocutors – that is, actors who were absent, but whose responses had to be anticipated (committees providing ethical oversight, NHS trust managers and frontline workers and members of the scientific community). Much of each meeting was taken up in navigating these demands, based on senior researchers’ previous experience and intimate knowledge of the Trust's organizational culture. Furthermore, this navigation had a very particular steer: to pre‐empt demands of both funder and grant holder (as funder's representative). Consequently, the contributions that PPI representatives were able to make were governed by these anticipated demands.

This orientation towards those conjured interlocutors was enacted through agendas and minute taking. Initially, agendas were produced by one of the early career researchers (ECRs) and itemized the different projects. Over time, an administrator was assigned and agendas began to mirror the templates of regular reports to the grant holder and funder, with items on ‘deliverables’ (eg ‘publications’, ‘collaboration with industry’). While the funder required annual progress reports, the grant holder had installed an additional internal reporting apparatus to ensure that the orientation towards funder demands was embedded in all activities. The material dimensions of the meetings’ spatial logic shaped its envisaged social relations via the installation of many absent others; the meetings’ orchestration of time, meanwhile, oriented the team towards the kinds of ‘deliverables’ imagined as capable of satisfying this apparatus.

In this context, imagined actors’ responses were frequently rehearsed in a way which suggested preferred courses of action: for example, senior clinicians often cautioned against certain protocol amendments, as these might occasion delays and derail timelines (eg ‘[the Research] Ethics [procedure] is a “can of worms”’ – B. senior clinician). On another occasion, the agenda item on ‘collaboration with industry’ elicited some discomfort from both PPI representatives and junior researchers, who expressed reservations on potential involvement with the pharmaceutical industry. While such involvement was not in fact undertaken, senior clinicians stepped in to remind of funder expectations (‘remember in [GRANT NAME] you get brownie points for collaboration with industry […] it's health *and* wealth’ – D. senior clinical academic). These examples illustrate how a temporal logic of deliverables – in which a projected future (one governed by a funder's demand) and installed through a reporting apparatus (the grant holder's conjuring of that demand) – disciplined and oriented actions of members of the team, while bracketing off possibilities for PPI representatives’ contributions in the present.

#### 4.1.2 Distancing oneself from conjured others

Researchers often performed a distancing from, or enacted a gentle mockery of, the demands placed upon them by various powerful agents. One senior clinician routinely baulked at the grant acronym, stating that the purpose and inner workings of the grant, and therefore funder intentions, were somewhat mystifying, a statement with which the assembled more junior researchers jokingly concurred. Statements such as these performed impotence in relation to powerful, and absent, partners (eg funder, clinical directors) and served to create complicity between PPI representatives and other team members as both lacking executive authority and having a partial perspective rather than the imagined ‘bird's‐eye view’ of the authoritative other. Furthermore, researchers often declared frustration over requirements for internal reports which, it was implied, detracted from the business at hand, that is, team projects. However, this distancing disavowed that the business at hand in all meetings, regardless of the timing of the reports, was in fact conjuring the demands of absent interlocutors, a process enacted through the circulated documents and their structuring of time and team orientation.

### 4.2 Maintaining the appearance of inclusion

Although the conjuring of powerful actors constrained the PPI representatives’ contributions, in interviews and informal discussions, both representatives contrasted the meetings favourably with other experiences, which one of them described as ‘tokenistic’. They repeatedly presented the meeting space as ‘ha[ving] quite an open and inclusive feel … [without] a feeling of hierarchies’ (M., PPI representative), and remarked on team members’ attentiveness: ‘I was listened to and … my input was felt to be worthwhile’ (W., PPI representative). Thus, an appearance of inclusion was maintained, which obscured the inflexibility of the meeting steer.

#### 4.2.1 Having a place at the table

Following both representatives’ suggestion, a ‘PPI item’ was included at the end of the agenda from the first meeting and remained there for all meetings, its presence legitimizing the representatives’ place at the table even in their absence. Furthermore, the architecture of the meeting, as well as the performances within it, served to cement a logic and rhetoric of inclusion, which indicated meaningful dialogue with PPI representatives. Team members were particularly courteous with them, respectfully listening whenever they spoke. Notably, since both representatives had existing relationships with team members, meetings acted as opportunities for transactions beyond the scope of the projects: in one meeting, the service user representative was reminded she needed to sign off on a group paper in which she was co‐author, while the carer representative was invited to give a talk to a committee another clinician chaired. Conversely, representatives made use of clinicians’ knowledge of NHS processes and research findings to support their own or their network's needs.

This performance of respect may have paradoxically served to deflect attention from the fact that PPI representatives were not in a position to challenge the conjuring of funder expectations. For example, one representative queried the use of nudges/prompts on a health checklist to encourage service users to seek regular physical health assessments. She argued that in caring for her sister, she already had to deliver on‐going prompts about medication, which her sister failed to recall. How would adding even more prompts help her sister become more autonomous? Wouldn't such prompts further intensify a carer's responsibilities instead? The chair listened attentively and suggested this might make for an interesting qualitative project – and then moved on to the next agenda item. This response had a double function. It offered the representative legitimization – by suggesting that such an insight could open up a new field for research (this *can* be another research project); at the same time, however, it removed these concerns from the present discussion into a potential future endeavour (this can be *another* research project). The possibility of this happening now and here was evacuated. The spatial‐temporal logic of the meeting ended up excluding the force of PPI even as it legitimated its epistemic potentiality.

#### 4.2.2 The affordance of explanatory scaffolding

Considerable explanatory scaffolding was set up to counter the PPI representatives’ infrequent contact with the research team: this consisted in regular project updates and emails (assiduously managed by one ECR), as well as on‐going, courteous explaining of NHS organizational habits, research processes and funder expectations, typically undertaken by the senior members of the team during the meetings themselves. This meant that, while some of the meeting time was dedicated to attending to PPI representatives’ assumed lack of knowledge, such attentiveness was enacted as an induction to the team's constraints – why things couldn't be done otherwise. Project updates performed a similar function: in clear language, they informed about project progress, while also presenting a smooth, simplified retrospective narrative to which the representatives were inevitably external. The updates referred to what had already happened and oriented the representatives’ oversight towards a closed past with which they could not negotiate, while the oversight performed by the senior clinicians referred to an anticipated future. It was as though the means of access to the projects (the clear account of their progress) was also a means of barring access. The role of the updates as a means of barring access was made visible when, on occasion, their account was challenged. For example, during one meeting, W. (PPI representative), having noticed that the update mentioned a paper under review (a key ‘deliverable’ for the team), challenged how ‘harm’ had been defined and measured therein. In response, researchers asserted the validity of their choices, first to W. and then to each other. Despite W. persisting with her challenge for several meetings, it was minuted only once:

[Researcher’s name] confirmed that [redacted] was measured as it is the most reliable data available. [Researcher] also assured the team of her confidence in the design used as the most robust method available. (Minutes of team meeting 12.12.2016)

The researcher's assurance re‐assured: it restored the integrity of the updates’ retrospective narrative in which questions of design had already been settled, thus rendering W.’s challenges out of time. The absence of W.’s further challenges from subsequent minutes reinforced and preserved the temporal logic of projects and agenda alike, proofing it against further disruptions.

### 4.3 Installing PPI as a constitutive ‘elsewhere’

The relationship between the PPI item, the representatives’ talk in the meetings, and ‘PPI’ as something that needed to be part of the studies remained unclear – despite the explanatory scaffolding, extensive transactions with the two representatives, and the on‐going presence of the PPI agenda item. In one meeting, SP observed the researchers trying to imagine how potential participants might react to being contacted by their mental health team – without turning to the PPI representatives. During a discussion on how service users use their mobile phones, a senior researcher turned to SP saying ‘PPI can help us here’ – even as the two representatives were present. Presumably, this was in the expectation that SP, as a researcher paid to investigate PPI, would suggest possibilities. In those meetings not attended by the representatives, what was referred to as ‘doing the PPI’ item became an opportunity for ECRs to report back on any PPI‐related activities they may have participated in.

PPI, then, cohered precisely as a site of uncertainty – neither moored to particular aspects of the study nor securely associated with specific interlocutors or actions. At the same time, SP glimpsed a related phenomenon: team members would typically mention to SP that PPI *had already taken place* in earlier stages (prior to SP’s observations) or was planned *for a later stage* (eg there would be user‐led evaluations on the feasibility of the physical health intervention; service users had worked with the team to evaluate an intervention on substance misuse). These sites for PPI were either temporally out of sync with the present project or contemporaneous, yet occurring in parts of studies for which SP had no ethics clearance.

After several occurrences of this, SP suspected they had not simply missed PPI by choosing to observe the wrong meetings, but that the location for PPI was a *constitutive elsewhere*. It was comparable to the ‘*other research project’* which a senior team member had invoked when one representative challenged the premises of the health checklist. This suspicion was given credence by the visualization of PPI in a PowerPoint presentation on the workstream delivered to the grant governing body. One slide was a graph with the linked projects represented as individual columns. A double‐headed arrow ran beneath them, separate from the columns and captioned ‘PPI’. The arrow’s purpose was to indicate that PPI was present throughout all studies but it was not clear what that presence consisted in. That lack of specificity in content and location thus manifested the ‘everywhere’ as nowhere: running across the projects but hard to locate and specify, a constitutive part yet spatially and temporally apart from the rest.

### 4.4 Acculturating junior researchers: meetings as sites of apprenticeship

In installing the spatial and temporal logics of deliverables – in which PPI was a part, apart – meetings also served to acculturate junior members into organizational cultures. Through practices of steering as course correction, ECRs learned to hone and redirect their energies towards the ‘correct’ set of imagined interlocutors. In the observed projects, regular reports to both senior clinical academics and PPI representatives testified to ECRs’ capacity to broker and mediate partnerships across managers, administrators, IT services and care workers. Initially tasked with the writing of agendas and minutes, R., the ECR managing several projects, had to adjudicate what was recordable and actionable, how a meeting was to be recast as signal and noise, recorded and forgotten. By the end, and as a result of such repeat performances, the ECR had internalized the narrative through which the research project will have been completed according to protocol and the constraints of various regulatory systems. The same ECR also wrote the updates to PPI representatives, their retrospective clarity and abstraction consolidating his identity as one belonging within an institutional space.

This ECR – a strong proponent of PPI – told SP, when interviewed in early months of the project, of his ambitious plans for how involvement could be folded into these studies. With HiM, for example, he discussed a process evaluation to be led by peer researchers, as well as the potential involvement of study participants themselves in a steering committee of a larger study:

‘if the project is feasible, we’d like to do a bigger project, and what I’d really like is that we have some of the people that have gone through the intervention to work with us on that’ R. – ECR

However, neither the evaluation nor the subsequent scale‐up proposals involved collaborations with service users. The ECR, discussing the same study with SP in the final workshop, with the assembled team members nodding in agreement, suggested that timelines, bureaucracy and other pragmatic reasons [notably that ‘can of worms’ ethics committee] had scuppered his initial plans. He concluded: ‘we [researchers] must get smarter’ so as to set up PPI in a more meaningful way in future studies.

This narrowing of the horizon of what was possible was an effect of learning how to work within the normative temporal and spatial coordinates of research environments and their imagined demands. In internalizing these coordinates, the ECR had also inadvertently internalized that constitutive elsewhere in which to locate PPI: it had been relocated to that ‘other research project’ – an anticipated future which might never arrive.

## 5 DISCUSSION: FROM THE JIGSAW TO THE LIMPET

In the final workshop, one participant said:

‘The question is whether you see [the PPI role] as a kind of *limpet on a ship*. So the ship is [research] and [PPI representative] comes and plonks itself on it and has very little contact with what the researchers are doing … you are just stuck on the side of something and people are very polite and they ask your opinion but basically they are doing what they want’ (D – senior clinical academic, italics added)

While the figure of the missing jigsaw piece is commonly used to describe PPI, our ethnography suggests that the figure of the ‘limpet on a ship’ may be a more accurate description of PPI within grant‐driven, applied health research projects. Through attending to the choreography of oversight meetings as social form, we have witnessed their installation of absent partners that matter and their subjection to a reporting apparatus that privileges certain activities and forecloses other kinds of action.^29^ Here, we build on anthropological research on meetings which has demonstrated how meetings serve to establish and situate actors within ‘a network of relations’ such that the technologies deployed within a meeting become crucial for demonstrating partnerships and participation.^30^ To deploy Renedo and Marston's tripartite division of the material, temporal and social dimensions of participatory spaces^22^: the material aspects of the meetings – the tools and techniques used to frame them – not only structured their temporal orientation, but in doing so, shaped their social relations. The conjuring of imagined social actors external to the meeting shaped the social relations able to unfold in the meetings themselves. In such meetings, PPI was an entity both external to, and temporally disjunct from, health research: more ‘limpet’ than missing puzzle piece. This argument is distinct from a claim that PPI came too late on the scene – that the representatives’ role was defined by the researchers and that they were not present early enough to have an effect on the projects’ design. Our findings suggest instead that the ‘performative governance’^31^ of research results in PPI never exactly being ‘in place’ or ‘in time’ – but rather repeatedly imagined as about to take place or having already taken place. A place on/at the (time)table enabled the PPI representatives to speak, yet at the same time rendered their voices supplementary or ‘out of time’ as regards the business at hand. Thus, PPI representatives’ interventions, even when validated, remained at odds with a meetings logic oriented towards a particular imagined future. The architecture and rituals of inclusion through which PPI representatives were made to feel welcome served, paradoxically, to distract PPI representatives from their exclusion from the chorus of imagined interlocutors. At the same time, PPI representatives’ presence provided an alibi for citizen/patient involvement – providing assurance that the team was working in an ethical manner in relation to an imagined community of lay people/patients/beneficiaries, while keeping, in effect, that community perpetually displaced from the business at hand.

The figure of the jigsaw, in visualizing a set of interlocking types of expertise, claims a parity between the PPI representative and forms of certified expertise. In so doing, the jigsaw flattens out the power relations that organize knowledge^32^, ^33^ – power relations which research on PPI, especially work by service user researchers, has extensively demonstrated.^21^, ^42^ Rather than a stable, two‐dimensional plane, our study demonstrates how collaboration comprises complex choreographies, which reduced the likelihood of direct contestation or acknowledgement of who wielded epistemic authority in this space.^43^ Our study intervenes theoretically and substantively in some dominant interpretative frameworks governing research on PPI. While there have been ethnographies of PPI, these have, for the large part, not engaged substantially with the anthropological literature on meetings. The logic of the part, apart – one of our key findings – became visible not only through our commitment to ethnographic methods, but through an approach that focused on meetings’ ‘conjured contexts’.^24^ These contexts demanded that PPI be simultaneously everywhere and nowhere (cf. Madden and Speed's characterization of the operations of PPI as an ‘empty signifier’).^44^ Furthermore, our findings draw attention to the professionalization of *researchers* – an under‐examined issue in research on PPI, even as such research features extensive discussion of the liminal position of PPI contributors and the consequences of *their* potential professionalization.^45^, ^46^, ^47^, ^48^ Indeed, the routine use of the term ‘researchers’ or ‘academics’ in PPI research obscures the complex professional power relations structuring academic work and applied research in particular – although some work on this is now emerging.^49^ In discussing the acculturation of the junior researcher, we show how meetings produce researcher identities by honing ECRs’ institutional fluency through their engagement with technical apparatuses and absent/imagined actors. This helps ensure the social reproduction of the normative research enterprise – and likely narrows the horizon of the possible in relation to the potential, future contributions of PPI.

Our research, in concert with existing studies, demonstrates how difficult it is for PPI representatives to make epistemic contributions within committees. But we go further, in arguing that principles commonly thought to optimize such epistemic contributions (eg clear feedback, respect, significant good will),^2^, ^33^ or indeed, the ability of PPI representatives to move within professional spaces as engaged actors,^22^ might tend, in fact, to detract attention from structural barriers to substantive contributions within the architecture that the meetings themselves install. As Komporozos‐Athanasiou and colleagues have argued, attempts to encourage 'active citizen spaces' of PPI allow those citizen participants ‘little room for re‐writing the rules of participation’.^11^ Our findings also problematize current investments in operationalizing such ‘active citizen spaces’ through measuring instruments which seek to define good PPI practice in terms of levels or standards of participation. One unsettling implication of our study is that attempts to optimize or standardize PPI within health research projects might end up further displacing PPI both spatially and temporally from the very sphere of action where research takes place.

## 6 CONCLUSION

We contribute to the literature on epistemic politics in PPI^45^, ^47^, ^50^, ^51^, ^52^, ^53^ by arguing that it is only by attending to the spatio‐temporal logics through which PPI representatives are embedded within research projects that we can understand the epistemic challenges they face. We recommend further research on the spatio‐temporal logics of meetings to better understand how they might contribute to making PPI essentially supernumerary to the requirements that constitute research – its hull, as it were. We are left with what it might mean to conjure alternative means of choreographing collaborative research involving PPI. Such alternatives would demand ensuring that the potential for PPI to be an active agent within them is not evacuated.^56^ Within a logic of deliverables and partners who matter, PPI is constitutively out of sync and out of place. Finding alternatives might mean envisaging ways of breaking apart and remaking the hull.

## 7 LIMITATIONS

SP was the sole ethnographer. We attempted to increase validity of our findings through having both authors involved in data interpretation and running a workshop presenting preliminary findings, in which participants reflected back to SP their assessments of PPI in the study, which fed into our interpretations here. SP had further interactions with some researchers in the study, as they were employees of the same institution. These interactions added to the familiarization with and ‘thickness’ of the data but also potentially created blind‐spots, as SP takes this organizational culture for granted in their own working lives. Anthropological studies of meetings often note the ethnographer's own acculturation through meetings can be a limitation but also a productive challenge which can give depth to observations.^57^ Finally, we acknowledge our data relate to a particular instantiation of PPI: the PPI representatives had been invited to join a steering committee to consult on projects already in progress when they arrived. However, such late arrivals are the case in many enactments of PPI, while the steering of projects through ‘delivering’ and ‘reporting’ in relation to a funder is an integral part of applied health research in general.

## 8 NOTE

We recognize that the term ‘PPI representative’ can be seen as a problematic choice. There are considerable debates attached to the use of the alternatives – ‘survivor’, ‘service user/carer’, ‘lived experience contributor’ and ‘lay advisor’. When asked, the two participants had different preferred terms; therefore, ‘PPI representative’ was used as a compromise.

## CONFLICT OF INTEREST

SP declares that while undertaking the study presented here, they had some institutional contact with some of the participants in the study. SP shared a funder and a grant with some of the participants during the study. FC declares no conflict of interest.

## PATIENT OR PUBLIC CONTRIBUTION

Both authors contributed to the writing of the paper. Both authors are service user researchers, and so there was substantial patient involvement in the design, conduct, analysis and interpretation of the study. Additionally, the protocol for the study was presented and discussed at the Exchange Network, a mixed group of patients, carers, researchers and NHS workers set up through the Collaboration for Leadership in Applied Health Research and Care [CLAHRC] North West London, in June 2015. Preliminary findings from the study were presented to the team members who are the focus of the ethnography – and who include two PPI representatives (one a patient, the other a carer); their contributions fed back into the interpretation and analysis of findings presented here. Dr Angela Sweeney, a service user academic, read a complete draft of the manuscript and gave extensive comments and feedback.

## Data Availability

The data that support the findings of this study are available from the corresponding author upon reasonable request.
